# National trends in patient characteristics, interventional techniques and outcomes of endovascular treatment for acute ischaemic stroke: Final results of the MR CLEAN Registry (2014–2018)

**DOI:** 10.1177/23969873251334271

**Published:** 2025-05-02

**Authors:** Wouter H Hinsenveld, Josje Brouwer, Sanne J den Hartog, Agnetha Bruggeman, Manon Kappelhof, Ivo GH Jansen, Maxim JHL Mulder, Kars CJ Compagne, Robert-Jan B Goldhoorn, Hester Lingsma, Geert Lycklama à Nijeholt, Rob AR Gons, Lonneke FS Yo, Maarten Uyttenboogaart, Reinoud Bokkers, Bart H van der Worp, Rob H Lo, Wouter Schonewille, Paul Brouwers, Tomas Bulut, Jasper MM Martens, Jeannette Hofmeijer, Boudewijn AAM van Hasselt, Heleen den Hertog, Sebastiaan F de Bruijn, Lukas C van Dijk, Marianne A van Walderveen, Marieke Wermer, Hieronymus Boogaarts, Ewoud J van Dijk, Julia H van Tuijl, Issam Boukrab, Tobien AHCML Schreuder, Roeland Heijboer, Anouk D Rozeman, Ludo FM Beenen, Alida A Postma, Albert J Yoo, Stefan D Roosendaal, Jeannette Bakker, Adriaan CGM van Es, Sjoerd Jenniskens, Ido Remy van den Wijngaard, Menno Krietemeijer, René van den Berg, Joseph CJ Bot, Sebastiaan Hammer, Marieke Sprengers, Frederick Jan Anton Meijer, Miou S Koopman, Elyas Ghariq, Auke PA Appelman, Anouk van der Hoorn, Marc P van Proosdij, Bas FW van der Kallen, Olvert A Berkhemer, Jeroen E Markenstein, Eef J Hendriks, Jo PP Peluso, Christiaan van der Leij, Lucas Smagge, Saman Vinke, Sjoerd Pegge, Wouter Dinkelaar, Jan Albert Vos, Jelis Boiten, Inger de Ridder, Jonathan Coutinho, Bart J Emmer, Pieter Jan van Doormaal, Bob Roozenbeek, Yvo BWEM Roos, Charles BLM Majoie, Diederik WJ Dippel, Aad van der Lugt, Wim van Zwam, Robert van Oostenbrugge

**Affiliations:** 1 Department of Neurology, Maastricht University Medical Center+ and School for Cardiovascular Diseases (CARIM), Maastricht, The Netherlands; 2 Department of Neurology, Amsterdam UMC Location University of Amsterdam, Amsterdam, The Netherlands; 3 Department of Neurology, Radiology, and Public Health, Erasmus University Medical Center, Rotterdam, The Netherlands; 4 Department of Radiology and Nuclear Medicine, Amsterdam UMC location University of Amsterdam, Amsterdam, The Netherlands; 5 Nicolab B.V., Amsterdam, The Netherlands; 6 Department of Radiology and Nuclear Medicine, Erasmus MC University Medical Center, Rotterdam, The Netherlands; 7 Department of Public Health, Erasmus MC, University Medical Center, Rotterdam, The Netherlands; 8 Department of Radiology, Haaglanden MC, The Hague, The Netherlands; 9 Department of Neurology, Catharina Hospital, Eindhoven, The Netherlands; 10 Department of Radiology, Catharina Hospital, Eindhoven, The Netherlands; 11 Department of Neurology, University Medical Center Groningen, Groningen, The Netherlands; 12 Department of Radiology, Medical Imaging Center Groningen, University Medical Center Groningen, Groningen, The Netherlands; 13 Department of Neurology and Neurosurgery, Brain Center, University Medical Center Utrecht, Utrecht, The Netherlands; 14 Department of Radiology, University Medical Center Utrecht, Utrecht, The Netherlands; 15 Department of Neurology, Sint Antonius Hospital, Nieuwegein, The Netherlands; 16 Department of Neurology, Medisch Spectrum Twente, Enschede, The Netherlands; 17 Department of Radiology, Medisch Spectrum Twente, Enschede, The Netherlands; 18 Department of Radiology and Nuclear Medicine, Rijnstate Hospital, Arnhem, The Netherlands; 19 Department of Neurology, Rijnstate Hospital, Arnhem, The Netherlands; 20 Department of Radiology, Isala, Zwolle, The Netherlands; 21 Department of Neurology, Isala, Zwolle, The Netherlands; 22 Department of Neurology, HAGA Hospital, The Hague, The Netherlands; 23 Department of Radiology, HAGA Hospital, The Hague, The Netherlands; 24 Department of Radiology, Leiden University Medical Center, Leiden, The Netherlands; 25 Department of Neurosurgery, Radboud University Medical Center, Nijmegen, The Netherlands; 26 Department of Neurology, Radboud University Medical Center, Nijmegen, The Netherlands; 27 Department of Neurology, Elisabeth-TweeSteden ziekenhuis, Tilburg, The Netherlands; 28 Department of Radiology, Elisabeth-TweeSteden ziekenhuis, Tilburg, The Netherlands; 29 Department of Neurology, Atrium Medical Center, Heerlen, The Netherlands; 30 Department of Radiology, Atrium Medical Center, Heerlen, The Netherlands; 31 Department of Neurology, Albert Schweitzer Hospital, Dordrecht, The Netherlands; 32 Department of Radiology and Nuclear Medicine, Maastricht University Medical Center+, and MHeNs School for Mental Health and Neuroscience, Maastricht, The Netherlands; 33 Department of Radiology, Texas Stroke Institute, TX, USA; 34 Department of Radiology, Albert Schweitzer Hospital, Dordrecht, The Netherlands; 35 Department of Radiology, Radboud University Medical Center, Nijmegen, The Netherlands; 36 Department of Radiology and Nuclear Medicine, Amsterdam UMC location Vrije Universiteit Amsterdam, The Netherlands; 37 Department of Radiology, Noordwest Ziekenhuisgroep, Alkmaar, The Netherlands; 38 Division of Neuroradiology, Joint Department of Medical Imaging, Toronto Western Hospital, University Health Network, Toronto, ON, Canada; 39 Department of Radiology, University Hospital Leuven, Leuven, Belgium; 40 Department of Radiology and Nuclear Medicine, Maastricht University Medical Center+ and School for Cardiovascular Diseases (CARIM), Maastricht, The Netherlands; 41 Department of Radiology, Sint Antonius Hospital, Nieuwegein, The Netherlands; 42 Department of Neurology, Haaglanden MC, The Hague, The Netherlands; 43 Department of Neurology, Erasmus MC University Medical Center, Rotterdam, The Netherlands

**Keywords:** MR CLEAN, stroke, endovascular treatment, acute ischaemic stroke, large vessel occlusion, interventional techniques, patient characteristics, registry, reperfusion

## Abstract

**Introduction:**

Endovascular thrombectomy (EVT) procedures and workflow have evolved over the years. We examined trends in patient characteristics, EVT techniques and outcomes over 5 years in the Netherlands.

**Patients and methods:**

Data from the MR CLEAN Registry (2014–2018) were analysed, including patients treated with EVT for anterior circulation acute ischaemic stroke (AIS). Patients were grouped by year of inclusion except for the linear regression analysis where the inclusion date was used. Baseline predicted probability of poor outcome (modified Rankin Scale (mRS) score 3–6) was calculated using a validated prediction model. Primary outcome was mRS score at 90 days. Secondary outcomes included workflow times, EVT techniques, successful reperfusion (eTICI ⩾ 2B) and symptomatic intracranial haemorrhage (sICH). Time trends were analysed using multivariable regression models (adjusted common odds ratios (acOR) per year).

**Results:**

5193 patients were included. Median age increased (from 66 in 2014 to 74 years in 2018 [*p* < 0.001]). Proportion of patients with pre-stroke dependence (mRS ⩾ 3) increased from 2014 through 2018 (9% to 16%, *p* < 0.001). Baseline predicted probability of poor outcome did not change (60% vs 66%, *p* = 0.06). Over time, functional outcomes improved (acOR 1.14 per year, 95%CI: 1.09–1.20); mortality decreased (aOR 0.88 per year, 95%CI: 0.83–0.94). EVT under local anaesthesia increased (from 46% in 2014 to 70% in 2018; aOR 1.15, 95%CI: 1.10–1.22), as did use of direct aspiration (13%–36%; aOR 1.43, 95%CI: 1.35–1.53). Successful reperfusion became more frequent (aOR 1.32 per year, 95%CI: 1.25–1.40), despite needing more attempts (1 in 2014 vs 2 in 2018, aOR 0.93 per year, 95%CI: 0.89–0.98). Incidence of sICH remained unchanged (5% vs 5%, aOR 0.99 per year, 95%CI: 0.89–1.09). Time from emergency room to groin puncture reduced by 7 min per year (95%CI: 5–8).

**Discussion and conclusion:**

Enhanced workflow and increased EVT experience may have led to shorter time to treatment and more frequent successful reperfusion, with better functional outcomes over 5 years, despite treating older, more dependent patients.

## Graphical abstract

**Graphical abstract:**
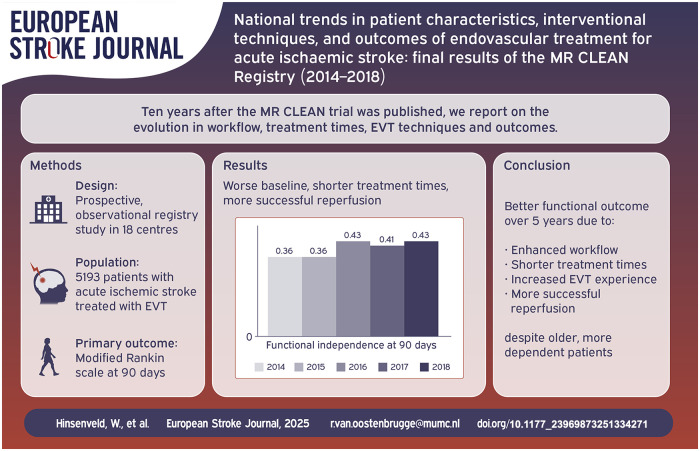

## Introduction

In 2014, The Multicenter Randomized Clinical Trial of Endovascular Treatment for Acute Ischaemic Stroke in the Netherlands (MR CLEAN) was the first randomized trial to show that endovascular treatment (EVT) within 6 h of symptom onset in patients with acute ischaemic stroke (AIS) caused by a proximal intracranial artery occlusion in the anterior circulation was effective and safe.^1^ These results were confirmed by other trials and EVT was implemented as standard treatment in many countries worldwide.^2^ Subsequently, the Dutch MR CLEAN Registry found consistent results of EVT in routine clinical practice.^3,4^ In 2018, two randomized controlled trials, DEFUSE 3 and DAWN, demonstrated benefit of EVT in selected patients with AIS up to 24 h of symptom onset.^5,6^ The wide implementation of EVT led to changes in workflow and EVT techniques, reduced time from onset to treatment and improved functional outcomes.^3,4^ There is also evidence that patients with multiple risk factors for unfavourable outcome can still benefit from EVT, for example, patients with pre-stroke dependence (modified Rankin Scale [mRS] score ⩾3), older patients, patients on antithrombotics and patients with large ischaemic cores.^7–11^ Ten years after the MR CLEAN trial was published, we report on the evolution in workflow, treatment times, EVT techniques and outcomes, including all centres also participating in the MR CLEAN trial. We hypothesized that over a 5 year period functional outcomes of patients with AIS due to a large vessel occlusion treated with EVT have improved, despite increased patient comorbidities and therefore increased probability of poor outcome.

## Methods

The MR CLEAN Registry is a multicentre, prospective, observational registry study in all 18 centres performing EVT in the Netherlands. Details on study design, data collection and study organization have been described previously.^3,4^ The central medical ethics committee of Erasmus MC University Medical Center, Rotterdam, the Netherlands, evaluated the study protocol and granted permission to carry out the study as a registry (MEC-2014-235).^3^ Further permission was obtained by the research board of each participating centre. In compliance with the General Data Protection Regulation, source data are not available for other researchers. Information about analytic methods, study materials and scripts of the statistical analyses are available from the corresponding author on reasonable request.

### Patients

We included adult patients treated with EVT between March 16, 2014, and December 31, 2018, with a proximal intracranial arterial occlusion of the anterior circulation (intracranial carotid artery [ICA, ICA-T], middle cerebral artery [MCA, M1/M2]) or anterior (A1/A2) cerebral artery), demonstrated by computed tomography angiography (CTA). In line with current daily practice, we included patients up to 24 h after symptom onset or last known well.

### Imaging, intervention and complications

EVT was defined as receiving arterial puncture with the intention to treat patients with an endovascular treatment method. The method of intervention and type of device used were left to the preference of the interventionist (i.e. with direct aspiration, stent retrievers, with or without use of intra-arterial thrombolytic agents, or any combination thereof). When intracranial access was achieved and Digital Subtraction Angiography (DSA) performed, but no mechanical thrombectomy was attempted (either due to spontaneous or alteplase-induced reperfusion or due to distal clot migration), the intervention was defined as ‘DSA only’. The term ‘Catheterization only’ was used when no intracranial access could be obtained, usually due to tortuous vessels or aortic elongation.

An independent imaging core laboratory committee consisting of experienced radiologists and interventional neurologist assessed non-contrast enhanced computed tomography (NCCT) and CTA at baseline, DSA and follow-up NCCT or CTA. They were provided with information on the symptomatic side and performed procedure but were blinded to all clinical findings and outcomes. Collaterals at baseline were scored on CTA using a 4-point scale. A score of 0 indicates absence of collaterals, and a score of 3 indicates 100% filling of the affected vascular territory.^12^

We defined successful reperfusion as an expanded Treatment In Cerebral Ischemia (eTICI) score of ⩾2B (⩾50% filling of the affected vascular territory) on the final DSA run, and excellent reperfusion as eTICI 2C-3 (90%–100% filling of the affected vascular territory).^13^ When the final DSA run was incomplete, that is, was only made in one direction, the maximum eTICI score was 2A. DSA results with only one single image (‘single shots’) were scored as missing.

### Baseline variables

We assessed trends over time in the following baseline variables: age, sex, National Institutes of Health Stroke Scale (NIHSS) score at baseline, mRS score before stroke onset, risk factors for stroke (history of hypertension, diabetes mellitus, atrial fibrillation, myocardial infarction or dyslipidaemia), transfer from a primary (i.e. thrombolysis-only) stroke centre, use of intravenous thrombolysis (IVT), occlusion segment on CTA, collateral score, Alberta Stroke Program Early CT score (ASPECTS) and late window patients (time from onset to groin puncture > 360 min). Predicted probability of poor outcome 90 days after EVT (mRS ⩾ 3) was assessed using the MR PREDICTS validated prediction tool.^14^ Baseline data on age, NIHSS at baseline, pre-stroke mRS, ASPECTS at baseline, systolic blood pressure, IVT use, glucose, occlusion segment, collateral score and time to EVT were used for this prediction.

We additionally analysed the following interventional variables: use of direct aspiration as first-line thrombectomy attempt, number of EVT attempts, anaesthesia technique (local anaesthesia vs sedation or general anaesthesia).

### Outcomes

We investigated trends over time using the full scale non-dichotomized ordinal mRS at 90 days (range 14 days either way) as primary outcome measure. Secondary clinical outcomes were rate of functional independence (defined as a mRS 2 or lower) and mortality at 90 days. We analysed first door to groin puncture time for both the overall inhospital time-interval (i.e. from primary centre to groin puncture including transfer) and for the intervention centre door to groin puncture time. Further secondary outcomes were onset to groin puncture time, time from EVT groin puncture to first reperfusion, eTICI score post intervention for patients with an EVT attempt, symptomatic intracranial haemorrhage (sICH) and stroke progression. Door time was defined as entry into the emergency room of the first hospital (i.e. the referral hospital for transferred patients), sICH was defined as worsening of the clinical symptoms with a ⩾4-point increase, or worsening of the clinical symptoms with a ⩾2 point increase on one item on the NIHSS and intracranial haemorrhage on follow-up non-contrast enhanced computed tomography (NCCT) according to the Heidelberg Bleeding criteria.^15^ Stroke progression was defined as any neurological deterioration ⩾4-points on the NIHSS scale without intracranial haemorrhage on NCCT.

### Missing data

All baseline data are reported crude. Missing data were imputed using multiple imputation based on relevant covariates and outcomes. If successful reperfusion was not achieved during EVT, we used time of last contrast bolus injection as the final reperfusion time. Any mRS score of 0–5 at follow-up assessed within 30 days of symptom onset was considered invalid and was treated as missing if no later mRS assessment was available.

### Statistical analysis

Patients were grouped per calendar year based on inclusion date. Baseline characteristics were reported per year (2014–2018) using counts and percentages for categorical variables, and medians and interquartile ranges for continuous variables.

The trends in baseline variables were assessed using the Cuzick trend test, which is an extension of the Wilcoxon ranked sum test that additionally tests for a unidirectional increase or decrease in values between ordinal groups instead of only a difference.^16^ For primary and secondary outcomes we used regression models after imputation in order to assess shifts over time. Effects were reported as either odds ratios (OR) with 95% confidence intervals for categorical and ordinal variables, or as beta coefficients (β) in minutes for time variables. For the primary outcome (mRS at 90 days) we used the non-dichotomized scale and calculated a common odds ratio showing the odds of a 1 point shift towards better functional outcome per year of inclusion. All regression analyses used day of inclusion as a continuous variable, but all odds ratios and beta coefficients are displayed per year of inclusion. All regression models were adjusted for age, sex, NIHSS score at baseline, pre-stroke mRS score, IVT administration, use of anticoagulation, a history of stroke and baseline systolic blood pressure. All workflow time outcomes were additionally adjusted for referral status, that is, if patients were transferred from primary stroke centres or presented directly to the intervention centre. We performed a sensitivity analysis for successful reperfusion excluding all patients that had missing two directional final DSA runs.

All analyses were performed in STATA version 14.

### Role of funding source

Applied Scientific Institute for Neuromodulation (Toegepast Wetenschappelijk Instituut voor Neuromodulatie) played no role in trial design and patient enrolment, nor in data collection, analysis or writing of the article.

## Results

### Patient characteristics

**Figure 1.**
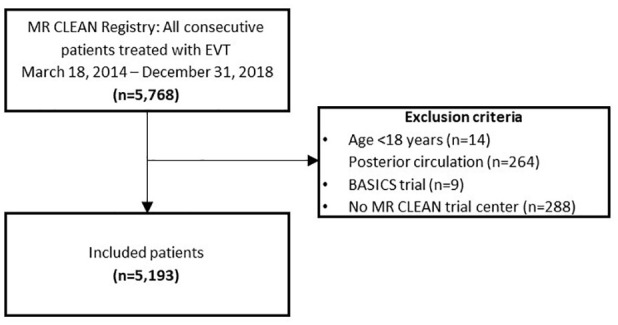
Flowchart study population.

**Figure 2.**
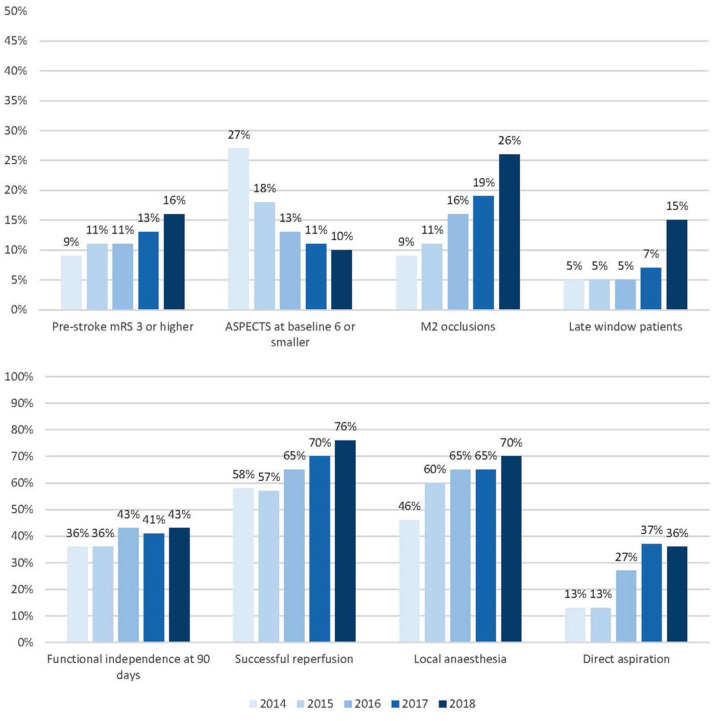
Evolution of baseline characteristics and outcomes over time.

We included a total of 5193 patients in this analysis (Figure 1). Each consecutive year, number of treated patients increased: from 193 patients in 10 months of 2014 (4% of total), 812 patients in 2015 (16%), 1131 patients in 2016 (22%), 1435 patients in 2017 (28%) to 1622 patients in 2018 (31%; Table 1). Throughout the years, median age of treated patients increased (median 74 years [IQR 65–81] in 2018, vs 66 years [IQR 55–74] in 2014, *p* < 0.001) and patients had a less severe neurological deficit at baseline (NIHSS: median 15 [IQR 9–19] in 2018, vs 16 [IQR 13–20] in 2014, *p* < 0.001), were more often dependent before stroke (16% in 2018% vs 9% in 2014, *p* < 0.001), and more often presented with a history of previous stroke (20% [2018] vs 15% [2014], *p* < 0.001) or hypertension (55% [2018] vs 39% [2014], *p* = 0.001; Figure 2). The proportion of patients with ASPECTS 6 or smaller decreased over time (27% [2014] vs 11% [2018], *p* < 0.001). Patients more often had distal occlusions during later years (26% M2 segment occlusions [2018] vs 9% [2014], *p* < 0.001). The percentage of patients with a good collateral score (>50% filling of occluded area) did not change over time (66% [2014] vs 61% [2018], *p* = 0.88). Patients were less often treated with IVT in later years, especially during 2018 (78% IVT treated in 2014 vs 70% in 2017 and 60% in 2018, *p* < 0.001). The predicted probability of poor outcome after EVT increased from 60% in 2014 to 66% in 2018. However, this did not show a statistically significant trend over time (*p* = 0.06).

**Table 1.** Baseline characteristics (*n* = 5.193).

Baseline Characteristic	2014 *n* = 193	2015 *n* = 812	2016 *n* = 1131	2017 *n* = 1435	2018 *n* = 1622	*p* Value for trend	Missing
Age (median, IQR)	66 (55–74)	71 (59–80)	71 (62–80)	73 (62–81)	74 (65–81)	<0.001	0
Age 80 or greater	24 (12%)	218 (27%)	301 (27%)	408 (28%)	503 (31%)	<0.001	0
Male sex	115 (60%)	420 (52%)	585 (52%)	734 (51%)	848 (52%)	0.46	0
NIHSS at baseline (median, IQR)	16 (13–20)	16 (12–20)	15 (11–19)	16 (10–19)	15 (9–19)	<0.001	72
NIHSS at baseline 10 or greater	168 (90%)	674 (84%)	880 (80%)	1121 (79%)	1197 (75%)	<0.001	72
Pre-stroke mRS 3 or higher	18 (9%)	89 (11%)	125 (11%)	178 (13%)	251 (16%)	<0.001	139
Previous medical history							
Previous stroke	28 (15%)	127 (16%)	195 (17%)	245 (17%)	325 (20%)	<0.001	39
Atrial fibrillation	32 (17%)	190 (24%)	265 (24%)	345 (24%)	397 (25%)	0.10	68
Myocardial infarction	25 (13%)	124 (16%)	163 (15%)	183 (13%)	232 (15%)	0.57	97
Diabetes mellitus	33 (17%)	146 (18%)	170 (15%)	237 (17%)	286 (18%)	0.72	31
Hypertension	74 (39%)	429 (53%)	594 (54%)	733 (52%)	885 (55%)	0.01	95
Dyslipidaemia	47 (25%)	240 (31%)	334 (31%)	421 (31%)	506 (32%)	0.11	208
Intravenous thrombolysis	149 (78%)	629 (78%)	851 (75%)	998 (70%)	958 (60%)	<0.001	30
ASPECTS at baseline (median, IQR)	8 (6–10)	9 (7–10)	9 (8–10)	9 (8–10)	9 (8–10)	<0.001	157
ASPECTS at baseline 6 or smaller	50 (27%)	137 (18%)	146 (13%)	158 (11%)	157 (10%)	<0.001	157
Good collateral score (>50% filling of occluded area)	124 (66%)	444 (59%)	604 (57%)	771 (57%)	946 (61%)	0.88	289
Occlusion segment on CTA						<0.001	218
ICA	6 (3%)	43 (6%)	62 (6%)	66 (5%)	89 (6%)		
ICA top	45 (24%)	181 (23%)	223 (21%)	259 (19%)	250 (16%)		
M1	120 (64%	450 (58%)	614 (56%)	783 (57%)	813 (52%)		
M2	16 (9%)	88 (11%)	178 (16%)	263 (19%)	397 (26%)		
Other (M3/A1)	0	10 (1%)	10 (0.9%)	6 (0.4%)	3 (0.2%)		
Transfer from primary centre	86 (45%)	446 (55%)	626 (55%)	784 (55%)	835 (52%)	0.57	3
Late-window patients (time to groin > 360 min)	10 (5%)	38 (5%)	57 (5%)	98 (7%)	237 (15%)	<0.001	90
Baseline predicted probability of mRS ⩾ 3 with EVT treatment (%, 95%CI)	60% (39–76)	63% (39–82)	63% (41–81)	61% (38–82)	66% (41–85)	0.06	1017
Local anaesthesia	76 (46%)	443 (60%)	687 (65%)	885 (65%)	1066 (70%)	<0.001	334
Use of direct aspiration^a^	23 (13%)	86 (13%)	245 (27%)	430 (37%)	474 (36%)	<0.001	203
Number of trombectomy attempts^a^ (median, IQR)	2 (1–3)	2 (1–3)	2 (1–3)	2 (1–3)	1 (1–3)	<0.001	0

NIHSS: National Institutes of Health Stroke Scale; mRS: modified Rankin Scale; IQR: interquartile range; ASPECTS: Alberta Stroke Program Early CT Score; CT(A): computed tomography (angiography); ICA: internal carotid artery; M1-3: middle cerebral artery first-third segment; A1: anterior cerebral artery first segment; EVT: endovascular treatment; CI: confidence interval.

Numbers are *N* (%) unless otherwise noted.

a In case an actual device attempt was made (*n* = 4421).

### Outcomes

**Figure 3.**
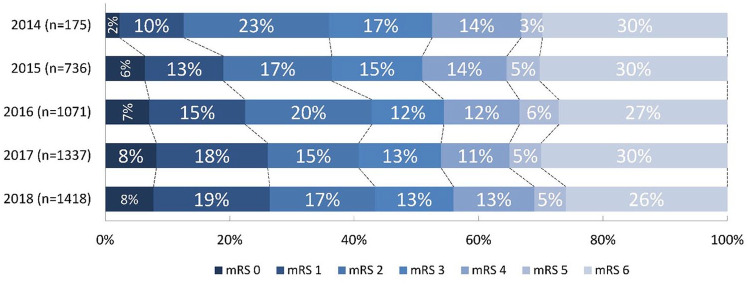
Modified Rankin Scale (mRS) score at 90 days. Stacked bar-chart showing percentages for each mRS score, with on the *x-*axis: percentage of patients with certain mRS, and on the *y*-axis: each year with number of patients. Adjusted common odds ratio for a shift towards a better outcome per year 1.14 (95%CI: 1.09–1.20).

mRS score at 90 days improved over the years with a shift towards better functional outcomes (acOR 1.14, 95%CI: 1.09–1.20) per year; Table 2, Figure 3). The proportion of functionally independent patients at 90 days increased each year (aOR 1.16, 95%CI: 1.09–1.23), leading to an absolute increase of 7% (36% in 2014 to 43% in 2018). Mortality decreased each year (aOR 0.88, 95%CI: 0.83–0.94) leading to an absolute decrease from 30% [2014] to 26% [2018]. The rate of sICH did not change (5% in both 2014 and 2018; aOR 0.99, 95%CI: 0.89–1.09) as did the rate of stroke progression (9% in 2014 vs 8% in 2018; aOR 0.94, 95%CI: 0.86–1.02).

**Table 2.** Primary and secondary outcomes.

Outcome	2014	2015	2016	2017	2018	Missing
*Clinical outcomes*						
mRS at 90 days (median, IQR)	3 (2–6)	3 (2–6)	3 (2–6)	3 (1–6)	3 (1–6)	456
Functional independence (mRS ⩽ 2) at 90 days	63 (36%)	268 (36%)	459 (43%)	545 (41%)	615 (43%)	456
Death < 90 days	52 (30%)	222 (30%)	290 (27%)	400 (30%)	368 (26%)	456
*Interventional outcomes*						
Successful reperfusion (eTICI ⩾ 2B)^a^	101 (58%)	393 (57%)	599 (65%)	825 (70%)	986 (76%)	166
*Complications*						
Symptomatic ICH	9 (5%)	52 (6%)	65 (6%)	91 (6%)	85 (5%)	0
Stroke progression	17 (9%)	83 (10%)	102 (9%)	122 (9%)	135 (8%)	0
*Workflow outcomes*						
Onset to groin puncture time in minutes (median, IQR)	208 (163–260)	215 (165–270)	195 (150–255)	181 (140–246)	189 (138–290)	90
First door to groin puncture time in minutes (median, IQR)	142 (107–189)	128 (96–167)	120 (88–152)	105 (76–137)	99 (71–133)	1238
Intervention centre door to groin puncture time in minutes (median, IQR)	92 (66–132)	71 (45–104)	57 (35–89)	51 (30–76)	51 (29–76)	493
First door to first CT scan time in minutes (median, IQR)	11 (8–18)	12 (7–17)	12 (7–19)	12 (7–19)	11 (6–17)	1395
Groin puncture to reperfusion time in minutes (median, IQR)	67 (44–100)	65 (40–90)	55 (36–80)	55 (36–80)	50 (33–75)	545

mRS: Modified Rankin Scale; CT: computed tomography; IQR: interquartile range; eTICI: extended thrombolysis in cerebral infarction; ICH: intracranial haemorrhage.

Numbers are *n* (%) unless otherwise noted.

a In case an actual device attempt was made (*n* = 4421).

### Interventional aspects

Use of local anaesthesia only, as compared to conscious sedation or general anaesthesia, increased from 46% in 2014 to 70% in 2018 (*p* < 0.001; Table 2). Contact aspiration as first line treatment increased over time (36% [2018] vs 13% [2014], *p* < 0.001) and there was a decrease in the number of thrombectomy attempts before successful reperfusion or end of procedure from a median of 2 attempts in 2014 to 1 in 2018 (*p* < 0.001). Successful reperfusion (eTICI ⩾ 2B) increased with an aOR of 1.32 per year (95%CI: 1.25–1.40) leading to an absolute improvement from 58% in 2014 to 76% in 2018.

### Workflow times

A total of 440 patients (8.5%) were treated in the late time window (time from onset to groin puncture > 360 min). The number of patients treated in the late window increased over time (5% in 2014, 2015, 2016, 7% in 2017, vs 15% in 2018, *p* < 0.001). An average of 7 min improvement per year (95%CI: 5–8; Table 3) in time of first emergency department entry to time of groin puncture was achieved leading to an absolute improvement of 43 min from 142 min in 2014 to 99 min in 2018. Treatment time from groin puncture to successful reperfusion was reduced by 3 min per year (95%CI: 2–4) and decreased from 67 min in 2014 to 50 min in 2018. The percentage of patients transferred from primary stroke centres to intervention centres for treatment showed no clear trend (45% in 2014 vs 52% in 2018, *p* = 0.57).

**Table 3.** Time trends in primary and secondary outcomes.

Outcome	Unadjusted (c)OR per year	Adjusted (c)OR per year
*Clinical outcomes*		
mRS at 90 days	1.08 (1.04 to 1.13)	1.14 (1.09 to 1.20)
Functional independence (mRS ⩽ 2)	1.08 (1.03 to 1.13)	1.16 (1.09 to 1.23)
Death <90 days	0.95 (0.90 to 0.99)	0.88 (0.83 to 0.94)
*Interventional outcomes*		
Local anaesthesia	1.18 (1.12 to 1.24)	1.15 (1.10 to 1.22)
Use of direct aspiration^a^	1.38 (1.30 to 1.46)	1.43 (1.35 to 1.53)
Number of thrombectomy attempts^a^	0.92 (0.88 to 0.96)	0.93 (0.89 to 0.98)
Successful reperfusion^a^	1.30 (1.23 to 1.37)	1.32 (1.25 to 1.40)
*Complications*		
Symptomatic ICH	0.98 (0.89 to 1.08)	0.99 (0.89 to 1.09)
Stroke progression	0.93 (0.86 to 1.01)	0.94 (0.86 to 1.02)
*Workflow outcomes*	Unadjusted B in minutes per year	Adjusted B in minutes per year
Onset-to-groin time	10 (7 to 14)	4 (1 to 7)
Door-to-groin time	−7 (−8 to −5)	−7 (−8 to −5)
Intervention centre door to groin time	−7 (−8 to −5)	−8 (−9 to −7)
First door to first CT time	0 (−0.3 to 0.4)	−0.2 (−0.6 to 0.2)
Groin-to-reperfusion time	−3 (−4 to −2)	−3 (−4 to −2)

OR: odds ratio; CI: confidence interval; mRS: modified Rankin Scale; ICH: intracranial haemorrhage; CT: computed tomography.

Numbers are Odds Ratios (95%CI) for all non-workflow outcomes. Trend in mRS is shown as common Odds Ratio per year. For all workflow times, numbers are minutes (95% CI). For all regression analyses, missing baseline and outcome data have been imputed, see methods section. Regression analyses were adjusted for age, sex, NIHSS score at baseline, pre-stroke mRS, intravenous thrombolysis, history of anticoagulation use and previous stroke, systolic blood pressure and occlusion segment on computed tomography angiography. Additionally all non-time-intervals were adjusted for ER entry to groin puncture time.

a In case an actual device attempt was made (*n* = 4421).

## Discussion

In our study we described trends in patient characteristics, details on EVT procedure, and EVT outcomes, in patients treated with EVT in our nationwide, consecutive, prospective, observational cohort over a 5-year period from 2014 to 2018. Clinical, radiological and workflow outcomes improved, even though patients had worse baseline characteristics and were more often pre stroke dependent. Shorter time to treatment combined with more frequent successful reperfusion may have contributed to these better outcomes. Baseline predicted probability of poor outcome showed only a slight and non-significant increase throughout the years (from 60% to 66%, *p* = 0.06).

### Comparison to other studies

Eligibility criteria for EVT became broader throughout the years, especially after the publication of the DAWN and DEFUSE-3 trials.^5,6^ A recent study performed after the implementation of these trials showed that the proportion of EVT cases treated after 6 h from last known well varied widely across hospital sites in the United States, but clearly increased after 2018, which is in line with our results.^17^ Additionally, in 2018 the MR CLEAN LATE trial started in several hospitals in the Netherlands including functionally independent patients selected with CTA only for potential EVT beyond 6 h. The start of this trial might have limited the amount of late window patients in our dataset. However, we think the impact of this trial on inclusion in the MR CLEAN Registry will have been limited due to the exclusion criteria of the trial: patients who fulfilled the characteristics of included patients in DAWN and DEFUSE were excluded from MR CLEAN LATE and included in the Registry.^18^

Since EVT became standard practice, results of several other registries have been published. The German registry (GSR-ET) used similar inclusion criteria: patients were consecutively collected, choice of EVT technique was left to the preference of the interventionist, and data were collected as part of clinical routine.^19,20^ Most baseline characteristics were similar to our study. The GSR-ET had less pre-stroke dependent patients (pre stroke mRS of ⩾3 11% vs 13% in our study), but more patients with known hypertension (75% vs 53%). The number of patients for whom outcome data were available were however limited in GSR-ET (*n* = 2794, which is 42% of all included patients).^19^ Comparing patients for whom outcomes were available, patients in our study had slightly better outcomes: mRS 0–2 was 41% in our cohort versus 37% in GSR-ET. Mortality did not differ, although in the GSR-ET more patients had an sICH (13% <24 h vs 5% at 90 days). Unfortunately, our trend analysis over time on outcomes and patient predicted probability of poor outcome could not be compared with other registries, because no such analysis has been performed in these studies.

Other registries are mainly industry-driven, do not group their patients per year, were retrospectively collected, were non-consecutive in nature or do not have an overview article reporting on outcomes.^21–25^ In some of these registries, the samples may not be representative of the nationwide population, while the MR CLEAN Registry included almost all EVT-treated patients in the Netherlands. In the industry-driven registries, patients who were not able to give informed consent within 7 days after EVT were excluded, possibly leading to a considerable selection bias as patients with less than desirable outcomes were more likely to be excluded, as informed consent had to be obtained by relatives.^23^ Moreover, most industry-driven registries concern EVT with one particular device precluding trend analysis in technique and limiting comparison with our registry. In both our study and the GSR-ET, the used EVT technique was left to the preference of the interventionist. We demonstrated that over the years the used interventional techniques shifted towards direct aspiration as first treatment attempt which might partially explain the reduced number of device attempts and the increased proportion of patients in which successful reperfusion was achieved. Additionally, our data shows an increase in the use of local anaesthesia compared to general anaesthesia or conscious sedation. This is considerably different to the GRS-ET where 96% of all patients were given either conscious sedation or general anaesthesia. There is considerable heterogeneity in current literature which anaesthetic technique is better.^26–28^ The increasing use of local anaesthesia in our data might be explained by MR CLEAN trial data demonstrating better functional outcomes using local anaesthesia, and by potentially faster treatment times and easier implementation of local anaesthesia.^29^ Most industry-driven studies included patients from the United States, where in less densely populated areas onset of stroke to hospital times may be long. This limits comparison with our data from the Netherlands where the density of hospitals is high and distances between primary stroke centres and intervention centres are small.

Studies on the increase of patient comorbidity throughout the years in AIS patients treated with EVT are scarce. Several studies show that patients with pre-existing disability and/or dependency who undergo EVT more often have comorbidities and higher age and have an increased risk on unfavourable outcome.^7,30,31^ In our study, patients were less often functionally independent and were older at baseline when compared to the initial MR CLEAN trial, which suggests a broader range of patients was treated. A MR CLEAN Registry substudy demonstrated that older age was associated with an increased absolute risk of poor clinical outcome, but that the relative benefit of successful reperfusion seems to be higher in these patients.^8^ Moreover, only 7.7% of all treated patients in the MR CLEAN trial had an M2-occlusion, while in our study 26% of all patients had an M2-occlusion, suggesting that with increasing experience over the years, more distal occlusions are being treated. ASPECTS at baseline also increased meaning that over time smaller infarct cores were treated. This might be due to the increasing proportion of treated patients with distal M2 occlusions combined with the decreasing time from stroke onset to groin puncture, both leading to smaller infarct cores and therefore higher ASPECTS. Despite this, the predicted probability of poor outcome (which includes ASPECTS and time to groin puncture) showed a trend towards poorer predicted outcome. We therefore assume that outcomes have also become better throughout the years due to growing experience with increasing patient volumes – similar to experiences in the surgical fields, where increasing experience is associated with better outcomes.

### Limitations and strengths

The MR CLEAN Registry has several limitations which are applicable to the current study as well. First, the MR CLEAN Registry only included patients in whom arterial puncture was carried out with the intention of treatment with EVT. This might have introduced some selection bias, especially in the earlier years where EVT experience was less and expertise and EVT may have been withheld in more complex patients. Possibly there is also a selection for treatment centres; that is, patients with worse baseline characteristics were possibly more likely to be treated in more experienced centres.

Second, in 2018, patient inclusion in the Netherlands started for two major randomized controlled trials: the MR CLEAN NO IV^32^ and the MR CLEAN MED.^33^ Patients who were included in these trials, were not included in the MR CLEAN Registry (*n* = 159). This introduces a possible patient selection bias. However, these patients would have been treated without these trials as well, which suggests that impact on outcomes will be limited. Also, no selection was made based on assumed prognosis. Baseline characteristics of these trials were similar to baseline characteristics in our study. In both trials, patients with pre-stroke disability which interferes with the assessment of functional outcome at 90 days, that is, mRS > 2, were excluded. This may have led to an overrepresentation of patients with pre-stroke mRS > 2 in our study.

Third, it is difficult to measure whether baseline predicted probability of poor outcome has changed. We used the validated MR PREDICTS tool to predict probability of poor outcome.^14^ Predicted probability of poor outcome suggested a trend towards an increase of predicted probability of poor outcome over the years, even though this was not statistically significant. However, altogether this may reflect the treatment of more patients with increasingly unfavourable baseline characteristics, albeit not statistically significant in our study. Importantly, there is no real scale or metric that uses all these variables and combines them into an interpretable score.

Strengths of this study are the large number of patients in a nationwide real-time registry, showing results of EVT in a real-life clinical setting. Another important strength is the use of a trained and experienced imaging core laboratory, limiting information bias in imaging assessment. Earlier studies have shown that self-assessed eTICI scores tend to be higher when compared to core laboratory adjudicated eTICI scores.^34^ It is unclear how DSAs with an incomplete post-treatment angiogram were assessed in other registries.

## Conclusion

Enhanced workflow and increased EVT experience may have led to shorter time to treatment and more frequent successful reperfusion, with better functional outcomes over 5 years, despite treating older, more dependent patients. Baseline predicted probability of poor outcome showed only a slight and nonsignificant increase throughout the years.

## Supplemental Material

sj-docx-1-eso-10.1177_23969873251334271 – Supplemental material for National trends in patient characteristics, interventional techniques and outcomes of endovascular treatment for acute ischaemic stroke: Final results of the MR CLEAN Registry (2014–2018) Supplemental material, sj-docx-1-eso-10.1177_23969873251334271 for National trends in patient characteristics, interventional techniques and outcomes of endovascular treatment for acute ischaemic stroke: Final results of the MR CLEAN Registry (2014–2018) by Wouter H Hinsenveld, Josje Brouwer, Sanne J den Hartog, Agnetha Bruggeman, Manon Kappelhof, Ivo GH Jansen, Maxim JHL Mulder, Kars CJ Compagne, Robert-Jan B Goldhoorn, Hester Lingsma, Geert Lycklama à Nijeholt, Rob AR Gons, Lonneke FS Yo, Maarten Uyttenboogaart, Reinoud Bokkers, Bart H van der Worp, Rob H Lo, Wouter Schonewille, Paul Brouwers, Tomas Bulut, Jasper MM Martens, Jeannette Hofmeijer, Boudewijn AAM van Hasselt, Heleen den Hertog, Sebastiaan F de Bruijn, Lukas C van Dijk, Marianne A van Walderveen, Marieke Wermer, Hieronymus Boogaarts, Ewoud J van Dijk, Julia H van Tuijl, Issam Boukrab, Tobien AHCML Schreuder, Roeland Heijboer, Anouk D Rozeman, Ludo FM Beenen, Alida A Postma, Albert J Yoo, Stefan D Roosendaal, Jeannette Bakker, Adriaan CGM van Es, Sjoerd Jenniskens, Ido Remy van den Wijngaard, Menno Krietemeijer, René van den Berg, Joseph CJ Bot, Sebastiaan Hammer, Marieke Sprengers, Frederick Jan Anton Meijer, Miou S Koopman, Elyas Ghariq, Auke PA Appelman, Anouk van der Hoorn, Marc P van Proosdij, Bas FW van der Kallen, Olvert A Berkhemer, Jeroen E Markenstein, Eef J Hendriks, Jo PP Peluso, Christiaan van der Leij, Lucas Smagge, Saman Vinke, Sjoerd Pegge, Wouter Dinkelaar, Jan Albert Vos, Jelis Boiten, Inger de Ridder, Jonathan Coutinho, Bart J Emmer, Pieter Jan van Doormaal, Bob Roozenbeek, Yvo BWEM Roos, Charles BLM Majoie, Diederik WJ Dippel, Aad van der Lugt, Wim van Zwam and Robert van Oostenbrugge in European Stroke Journal
